# Supplementary material for: Representation of the spatio-temporal narrative of The Tale of Li Wa李娃传
Source: PLoS One. 2020 Apr 23;15(4):e0231529. doi: 10.1371/journal.pone.0231529 (PMC7180071; doi:10.1371/journal.pone.0231529)
Supplement: S1 File — (PDF) [file pone.0231529.s001.pdf]

太平廣記卷四百八十四　宋　李昉等　編

雜傳記

李娃傳

李娃傳

汧國夫人李娃長安之倡女也節行瓌竒有足稱者故監察御史白行簡為傳述天寶中有常州刺史滎陽公者畧其名字不書時望甚重家徒甚殷知命之年有一

子始弱冠矣雋朗有詞藻迥然不羣深為時輩推伏其父愛而器之曰此吾家千里駒也應鄉賦秀才舉將行乃盛其服玩車馬之飾計其京師薪儲之費謂之曰吾觀爾之才當一戰而霸今備二載之用且豐爾之給將為其志也生亦自負視上第如指掌自毘陵發月餘抵長安居于布政里嘗遊東市還自平康東門入將訪友于西南至鳴珂曲見一宅門庭不甚廣而室宇嚴邃闔一扉有娃方凭一雙鬟青衣立妖姿要妙絶代未有生

忽見之不覺停驂久之徘徊不能去乃詐墜鞭于地候其從者勑取之累眄于娃娃回眸凝睇情甚相慕竟不敢措辭而去生自爾意若有失乃密徵其友遊長安之熟者以訊之友曰此俠邪女李氏宅也曰娃可求乎對曰李氏頗贍前與通之者多貴戚豪族所得甚廣非累百萬不能動其志也生曰苟患其不諧雖百萬何惜他日乃潔其衣服盛賓從而往扣其門俄有侍兒啟扃生曰此誰之第耶侍兒不答馳走大呼曰前時遺策郎也

娃大悦曰爾姑止之吾當整粧易服而出生聞之私喜
乃引至蕭墻間見一姥垂白上僂即娃母也生跪拜前
致詞曰聞茲地有隙院願稅以居信乎姥曰懼其淺陋
湫隘不足以辱長者所處安敢言直耶延生于遲賓之
館館宇甚麗與生偶坐因曰某有女嬌小技藝薄劣欣
見賓客願將見之乃命娃出明眸皓腕舉步艷冶生遽
驚起莫敢仰視與之拜畢敘寒燠觸類妍媚目所未覩
復坐烹茶斟酒器用甚潔久之日暮鼓聲四動姥訪其

居遠近生紿之曰在延平門外數里冀其遠而見留也姥曰鼓已發矣當速歸無犯禁生曰幸接歡笑不知日之云夕道里遼濶城内又無親戚將若之何娃曰不見責僻陋方將居之宿何害焉生數目姥姥曰唯唯生乃召其家僮持雙縑請以備一宵之饌娃笑而止之曰賓主之儀且不然也今夕之費願以貧窶之家隨其粗糲以進之其餘以俟他辰固辭終不許俄徙坐西堂帷幙簾幌煥然奪目粧奩衾枕亦皆侈麗乃張燭進饌品味

甚盛徹饌姥起生娃談話方切詼諧調笑無所不至生曰前偶過鄉門遇卿適在屏間厥後心常勤念雖寢與食未嘗或捨娃答曰我心亦如之生曰今之來非直求居而已願償平生之志但未知命也若何言未終姥至詢其故具以告姥笑曰男女之際大欲存焉情苟相得雖父母命不能制也女子固陋曷足以薦君子之枕席生遂下階拜而謝之曰願以己為厮養姥遂目之為郎飲酣而散及旦盡徙其橐槖因家于李之第自是生屏

跡戢身不復與親知相聞日會倡優儕類狎戲遊宴囊
中盡空乃鬻駿乘及其家僮歲餘資財僕馬蕩然邇來
姥意漸怠娃情彌篤他日娃謂生曰與郎相知一年尚
無孕嗣常聞竹林神者報應如響將致薦酹求之可乎
生不知其計大喜乃質衣于肆以備牢禮與娃同謁祠
宇而禱祝焉信宿而返策驢而後至里北門娃謂生曰
此東轉小曲中某之姨宅也將憩而覲之可乎生如其
言前行不踰百步果見一車門窺其際甚弘敞其青衣

自車後止之曰至矣生下驢適有一人出訪曰誰曰李娃也乃入告俄有一嫗至年可四十餘與生相迎曰吾甥來否娃下車嫗迎訪之曰何久踈絶相視而笑娃引生拜之既見遂偕入西戟門偏院中有山亭竹樹葱蒨池榭幽絶生謂娃曰此姨之私第耶笑而不答以他語對俄獻茶果甚珍奇食頃有一人控大宛汗流馳至曰姥遇暴疾頗甚殆不識人宜速歸娃謂姨曰方寸亂矣某騎而前去當令返乘便與郎偕來生擬隨之其姨與侍

兒偶語以手揮之令生止于户外曰姥且殁矣當與某議喪事以濟其急奈何遽相隨而去乃止共計其凶議齋祭之用日晚乘不至姨言曰無復命何也郎驟往覘之某當繼至生遂往至舊宅門扃鑰甚密以泥緘之生大駭詰其隣人隣人曰李本税此而居約已周矣第主自收姥徙居而且再宿矣徵徙何處曰不詳其所生將馳赴宣陽以詰其姨日已晚矣計程不能達乃弛其裝服質饌而食賃榻而寢生恚怒方甚自昏達旦目不交

曉贊明乃策蹇而去既至連扣其扉食頃無人應生大呼數四有官者徐出生遽訪之姨氏在乎曰無之生曰昨暮在此何故匿之訪其誰氏之第曰此崔尚書第昨者有一人税此院云遲中表之遠至者未暮去矣生惶惑發狂罔知所措因返訪布政舊邸邸主哀而進膳生怨懣絶食三日遘疾甚篤旬餘愈甚邸主懼其不起徙之于凶肆之中綿綴移時合肆之人共傷嘆而互飼之後稍愈杖而能起由是凶肆日假之令執繐帷獲其

直以自給累月漸復壯每聽其哀歌自嘆不及逝者輒
嗚咽流涕不能自止歸則効之生聰敏者也無何曲盡
其妙雖長安無有倫比初二肆之傭凶器者互爭勝負
其東肆車轝皆奇麗殆不敵唯哀挽劣焉其東肆長知
生妙絕乃醵錢二萬索顧焉其黨耆舊共較其所能者
陰教生新聲而相讚和累旬人莫知之其二肆長相謂
曰我欲各閱所傭之器于天門街以較優劣不勝者罰
直五萬以備酒饌之用可乎二肆許諾乃邀立符契署

以保証然後閲之士女大和會聚至數萬于是里胥告于賊曹賊曹聞于京尹四方之士盡赴趨焉巷無居人自旦閲之及亭午歷舉輦轝威儀之具西肆皆不勝師有慙色乃置層榻于南隅有長髯者擁鐸而進翊衛數人於是奮髯揚眉扼腕頓顙而登乃歌白馬之詞恃其夙勝顧眄左右旁若無人齊聲讚揚之自以為獨步一時不可得而屈也有頃東肆長于北隅上設連榻有烏巾少年左右五六人秉翣而至即生也整衣服俯仰甚

徐申喉發調容若不勝乃歌薤露之章舉聲清越響振林木曲度未終聞歔欷掩泣西肆長為衆所誚益慙恥密置所輸之直于前乃潛遁焉四座愕眙莫之測也先是天子方下詔俾外方之牧歲一至闕下謂之入計時也適遇生之父在京師與同列者易服章竊往覩焉有老豎即生乳母壻也見生之舉措辭氣將認之而未敢乃泫然流涕生父驚而詰之因告曰歌者之貌酷似郎之亡子父曰吾子以多財為盜所害奚至是耶言訖亦

泣及歸瑩問馳往訪于同黨曰向歌者誰若斯之妙歟皆曰某氏之子徵其名且易之矣瑩凛然大驚徐往迫而察之生見瑩色動回翔將匿于衆中瑩遂持其袂曰豈非某乎相持而泣遂載以歸至其室父責曰志行若此污辱吾門何施面目復相見也乃徒行出至曲江西杏園東去其衣服以馬鞭鞭之數百生不勝其苦而斃父棄之而去其師命相狎暱者陰隨之歸告同黨共加傷歎令二人齎葦席瘞焉至則心下微温舉之良久氣稍

通因共荷而歸以葦筒灌勺飲經宿乃活月餘手足不能自舉其楚撻之處皆潰爛穢甚同輩患之一夕棄于道周行路咸傷之往往投其餘食得以充腸十旬方杖策而起被布裘裘有百結襤褸如懸鶉持一破甌巡于閭里以乞食為事自秋徂冬夜入于糞壤窟室晝則周遊廛肆一旦大雪生為凍餒所驅冒雪而出乞食之聲甚苦聞見者莫不悽惻時雪方甚人家外户多不發至安邑東門循理垣北轉第七八有一門獨啟左扉即娃

之第也生不知之遂連聲疾呼饑凍之甚音響悽切所
不忍聽娃自閣中聞之謂侍兒曰此必生也我辨其音
矣連步而出見生枯瘠疥厲殆非人狀娃意感焉乃謂
曰豈非某郎也生憤懣絕倒口不能言頷頤而已娃前
抱其頸以繡襦擁而歸于西廂失聲長慟曰令子一朝
及此我之罪也絕而復蘇姥大駭奔至曰何也娃曰某
郎姥遽曰當逐之奈何令至此娃斂容卻睇曰不然此
良家子也當昔驅高車持金裝至某之室不踰期而蕩

盡且互設詭計捨而逐之殆非人令其失志不得齒于人倫父子之道天性也使其情絶殺而棄之又困躓若此天下之人盡知為某也生親戚滿朝一旦當權者熟察其本末禍將及矣況欺天負人鬼神不祐無自貽其殃也某為姥子殆今有二十歲矣計其貲不啻直千金今姥年六十餘願計二十年衣食之用以贖身當與此子別卜所詣所詣非遙晨昏得以温凊某願足矣姥度其志不可奪因許之給姥之餘有百金北隅因五家税

一隙院乃與生沐浴易其衣服為湯粥通其腸次以酥乳潤其臟旬餘方薦水陸之饌頭巾履襪皆取珍異者衣之未數月肌膚稍腴卒歲平愈如初異時娃謂生曰體已康矣志已壯矣淵思寂慮默想曩昔之藝業可温習乎生思之曰十得二三耳娃命車出遊生騎而從至旗亭南偏門鬻墳典之肆令生揀而市之計費百金盡載以歸因令生斥棄百慮以志學俾夜作晝孜孜矻矻娃常偶坐宵分乃寐伺其疲倦即諭之綴詩賦二歲而

業大就海內文籍莫不該覽生謂娃曰可策名試藝矣
娃曰未也且令精熟以俟百戰更一年曰可行矣於是
遂一上登甲科聲振禮闈雖前輩見其文罔不斂衽敬
羨願友之而不可得娃曰未也今秀士苟獲擢一科第
則自謂可以取中朝之顯職擅天下之美名子行穢跡
鄙不侔于他士當礱淬利器以求再捷方可以連衡多
士爭霸羣英生由是益自勤苦聲價彌甚其年遇大比
詔徵四方之雋生應直言極諫科策名第一授成都府

叅軍三事以降皆其友也將之官娃謂生曰今之復子本軀其不相負也願以殘年歸養老姥君當結媛鼎族以奉蒸嘗中外婚媾無自黷也勉思自愛槩從此去矣生泣曰子若棄我當自剄以就死娃固辭不從生勤請彌懇娃曰送子涉江至于劒門當令我回生許諾月餘至劒門未及發而除書至生父由常州詔入拜成都尹兼劒南採訪使浹辰父到生因投刺謁于郵亭父不敢認見其祖父官諱方大驚命登階撫背慟哭移時曰吾

與爾父子如初因詰其由具陳其本末大奇之詰娃安在曰送某至此當令復還父曰不可翌日命駕與生先之成都留娃于劒門築別館以處之明日命媒氏通二姓之好備六禮以迎之遂如秦晉之偶娃既備禮歲時伏臘婦道甚修治家嚴整極為親所眷向後數歲生父母偕歿持孝甚至有靈芝產于倚廬一穗三秀本道上聞又有白鷰數十巢其層甍天子異之寵錫加等終制累遷清顯之任十年間至數郡娃封汧國夫人有四子

皆為大官其甲者猶為太原尹弟兄姻媾皆甲門内外隆盛莫之與京嗟乎倡蕩之姬節行如是雖古先烈女不能踰也焉得不為之歎息哉予伯祖嘗牧晉州轉戶部為水陸運使三任皆與生為代故暗詳其事貞元中予與隴西公佐話婦人操烈之品格因遂述汧國之事公佐拊掌竦聽命予為傳乃握管濡翰疏而存之時乙亥歲秋八月太原白行簡云出異聞集

太平廣記卷四百八十四
