# Supplementary figures and images for: Representation of the spatio-temporal narrative of The Tale of Li Wa李娃传
Source: PLoS One. 2020 Apr 23;15(4):e0231529. doi: 10.1371/journal.pone.0231529 (PMC7180071; doi:10.1371/journal.pone.0231529)

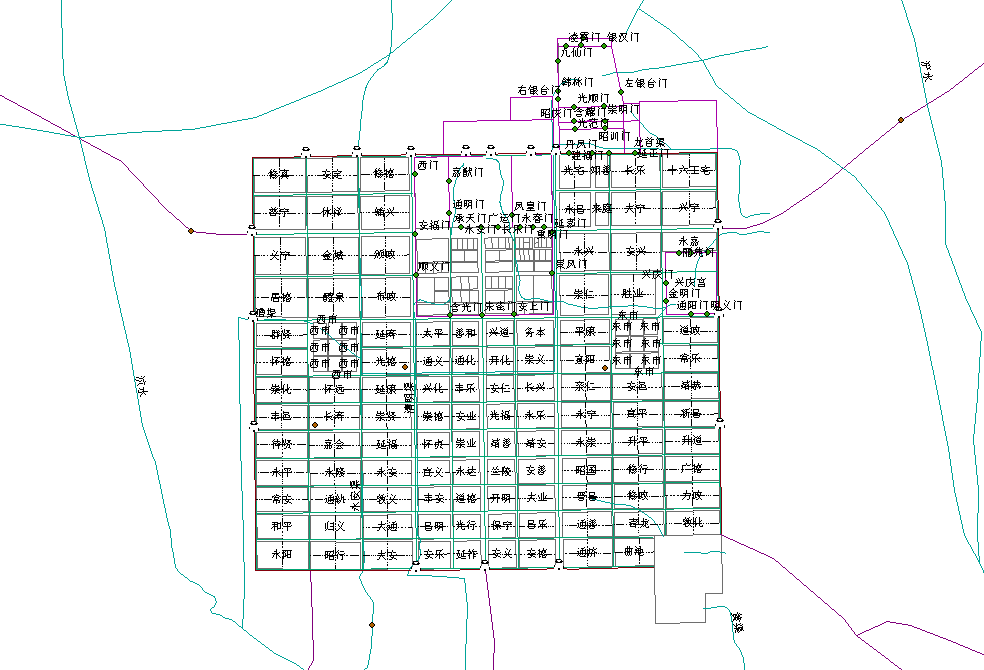

Supplement: S2 File — (ZIP) [file pone.0231529.s002.zip › Raster map of Tang Chang'an with location information/╠╞╢╝│ñ░▓.tif]
