# Supplementary material for: Representation of the spatio-temporal narrative of The Tale of Li Wa李娃传
Source: PLoS One. 2020 Apr 23;15(4):e0231529. doi: 10.1371/journal.pone.0231529 (PMC7180071; doi:10.1371/journal.pone.0231529)
Supplement: Attachment — Submitted filename: Ma2019_Representation.pdf [file pone.0231529.s012.pdf]

In this manuscript, *Representation of the patio-temporal narrative of The Tale of Li Wa*, Ma *et al.* provided a rather systematic study of the spatial and temporal structure of a traditional Chinese novel, the tale of Li Wa. To help modern readers to understand this classic, the authors have adopted several techniques and methods from data mining and network science. Although I find the study systematic and somewhat interesting, I have several questions regarding the "research question" of the paper and some technical details of the methods.

First, despite several methods have been adopted and hundreds of analysis have been made, it is very difficult to find a very clear statement of the research question in the paper. Is it about a demonstration of a new method? (It seems not, since all methods have been invented before). Or is it about using these analysis to support a known theory? Or the authors want to present some new insights from the analysis? The authors may have a clear answer to these questions and may have some deep insights in their mind, but it is very difficult (at least for me) to find the answer in the current presentation of the work. The current version is so over overwhelmed with different analysis without a clear thread to link them. Without a key message, it is also hard for the readers to tell the purpose of each analysis and understand the relationship between them. For example, what is the message the author want to convey in Fig. 17? What is the new insight of the plot? How could that deepen the reader's understanding of the book since it is like a confirmation that the main character is Zheng? By the way, is the color representing the communities learned from the network, did you use the same method shown in Fig. 21? If a different modularity method is adopted (see Modularity Q proposed by Girvan and Newman), whether the results shown in these two figures still hold?

In this sense, I suggest the author reorganize the narrative and structure of the paper, highlighting the research question and novelty of the paper. Rather than solely demonstrating the toolbox, I hope the author could provide the insight of each analysis.

Second, I also have a couple of comments about some technical details.

a) The assignment of the sentiment orientation (so) value seems a little bit arbitrary, since several words and characters could have ambiguous meaning and it is possible for different people have different feelings about the same word. Could the authors provide some cross-validations of the value assignment to make sure the value indeed present the basic expression? For example, maybe the authors could borrow tools from network science (as they did in the paper), and build a network of the sentiment expressions? (if two sentiment expressions are shown in one sentence or from one reading time snapshot, a link will be added between them) Of course, the author could try other alternative methods to do the cross-validation.

b) Can the authors provide the details of how do they measure the "stratum" in Fig. 18? Why the compass also have a color of light blue? Does that mean the person has shown there? The same issue for the lower panel of Fig. 13.
