# Supplementary material for: Representation of the spatio-temporal narrative of The Tale of Li Wa李娃传
Source: PLoS One. 2020 Apr 23;15(4):e0231529. doi: 10.1371/journal.pone.0231529 (PMC7180071; doi:10.1371/journal.pone.0231529)
Supplement: Attachment — Submitted filename: Response to Reviewers.doc [file pone.0231529.s013.doc]

# Responses to Reviewers

# 1. Response to the “lack of research” question pointed out by the editor

***Editor’s comment:*** *Both reviews (I also agree with them) pointed out the lack of research question in the current version. Please reshape the revised manuscript accordingly. In addition, please make sure to address the other issues raised by the reviewers in the revisions.*

Thanks for the editor's suggestion. We therefore rethought our research question and reorganized the entire paper with modifications. We propose a framework of digital models that integrate literary theory, providing contemporary readers with an additional opportunity to understand Chinese classical novels wrote in an almost unfamiliar language.

We show one of these possibilities under this framework through a knowledge model or narrative based on our team’s experience. It is the the gradually in-depth theme of "what-how-why" about the growth of the male protagonist hidden in the novel narritives. This knowledge model is gradually obtained through in-depth analysis and concatenation of various diagrams in the "Time-Space-time-Space" logical loop proposed in the manuscript.

## 1.1 Outline of the revised manuscript

People living in the digital age seem to have more difficulties when reading classical novels, in terms of obscure words, contextual difference and reading habits. This paper proposes a framework of digital models integrating literary theory to represent the narrative of a Chinese classic novel, The Tale of Li Wa, which has been diversely favored by literature and historians in the past approximately 900 years. To imitate reader’s reading psychological changes, we refer to three abstract spatial narrative theories: Bakhtin’s chronotopos (time–space) (Bakntin, 2002), which enlightens us to add historical context to the model; Zoran’s topographical-chronotopic-textual space (Zoran, 1984), which inspires us to reconstruct reader's reading experience in three dimensions; and Mitchell’s spatial metaphors (Mitchell, 1980), which indicates the core of reading lies in pursuit and acquisition of the changing pattern and meaning in the process of reading. In terms of methods, quantitative analysis including semantic analysis, sentiment analysis, spatial analysis, and network analysis are applied. Through the collation, analysis, and transmedia argumentation of information, normal readers can have the opportunity to choose their own model to represent and interpret the texts in their own perspective in the text’s knowledge graph. This paper presents one of these possible themes on illustrating the growth of male protagonist in our open framework of "Time-Space-time-Space", which opens up dialogues between computation and literature, diachronic and synchronic, reader and the author.

The time level shows the basic variables’ trajectories of read time, as well as the point of the story which is a growth story of the male protagonist when he first entering the capital Chang'an before servicing the bureaucracies. The space-time level unfolds various variables’ trajectories in the context of a two-dimensional urban space of read time, as well as the story’s details about how he lives and grows in the complex urban space of Chang'an. The space level represents the statistical pattern of mapping trajectories of different variables and a meaningful system indicating the social space of Chang'an City described in the novel. Through the integration of the chronotope of author, text, and reader, the understanding on the narrative, the physical background of Chang’an city, and the life of the residents of Chang’an are gradually profound within the looped spatio-temporal framework. We even find the primitive image of power control embodied in the story. From a different perspective, it helps us to have a better understanding on the author Bai Xingjian's bureaucratic career and his experience in Chang'an in the author's chronotope.

Our team explores the representation of spatio-temporal narrative of classical literature from a perspective of non-professional literature researchers (all majored in architecture, urban planning, and landscape architecture), and tries to gradually and opaquely interpret *The Tale of Li Wa* based on researchers’ personal experience. We must admit that this project is not a digital humanities project based entirely on specific content or assumptions. But such a process also proves the feasibility of our digital framework model from the perspective of reader's reading experience, which combines history with texts, the diachronic with synchronic. This research can be considered as a comprehensive exploration towards “narratives, experiences and geographical spaces”.

# 2. Responses to suggestion of minor revisions from Reviewer #1

Many thanks to Reviewer #1 for your deep understanding and appreciation of our paper.

## 2.1 Questions about References

***Reviewer’s comment:*** *The authors seem to summarize well the main research questions about this novel. However, I could not check the validity of many references for problems of linguistic understanding (there are in chinese). Readers therefore need to begin with an act of trust.
Note that readers may wish to have a short summary of statistical approaches to literature (without going into details). It would be possible, for example, to quote Franco Moretti's book, Graphs Maps Tree (among others).*

The electronic version of all references ***except*** the web references have been uploaded on github (<https://github.com/aayi/The-Tale-of-Li-Wa>). In view of some previous studies on *The tale of Li Wa*, I recommend that readers read its entry on Wikipedia（<https://en.m.wikipedia.org/wiki/The_Tale_of_Li_Wa>）. Actually there are a lot of previous literature about this novel, and I only select some typical ones to discuss in our paper. In addition, we supplemented our novelty based on these literature review.

More reference in Chinese can be found basically on CNKI (China National Knowledge Infrastructure, http://new.oversea.cnki.net/index/). Table 1 listed their url which may at least provide bilingual abstracts.

Table 1. Information About Chinese Reference.

| **Title** | **URL** | **DOI** |
| --- | --- | --- |
| [Cultural Interpretation of Chinese Narrative Literature] 中国叙事学的文化阐释 | <http://cn.oversea.cnki.net/kcms/detail/detail.aspx?DbCode=CJFR&dbname=CJFD2003&filename=GDMZ200303007&uid=WEEvREcwSlJHSldRa1FhdkJkVG1EWmxjVTFCT3ZkL1hKUGkvOVJBNk4ybz0=$9A4hF_YAuvQ5obgVAqNKPCYcEjKensW4IQMovwHtwkF4VYPoHbKxJw!!> |  |
| [History of Chinese Literature: Volumn on Sui]中国文学史话:隋唐五代卷 | <http://book.kongfz.com/162251/728624782/> |  |
| [Review of the Biography of Li Wa Research in the Past Ten Years] 近十年《李娃传》研究述评 | <http://new.oversea.cnki.net/KCMS/detail/detail.aspx?dbcode=CJFQ&dbname=CJFDLAST2017&filename=LNSW201701003&uid=WEEvREcwSlJHSldRa1FhdkJkVG1EWmxjVTFCT3ZkL1hKUGkvOVJBNk4ybz0=$9A4hF_YAuvQ5obgVAqNKPCYcEjKensW4IQMovwHtwkF4VYPoHbKxJw!!&v=Mjk1ODc0SDliTXJvOUZaNFI4ZVgxTHV4WVM3RGgxVDNxVHJXTTFGckNVUkxPZmIrUnZGeW5uVmJyT0tTUFllYkc=> | 10.13888/j.cnki.jsie(ss).2017.01.003 |
| [On the New Field of Narratology: space narrotology] 空间叙事学：叙事学研究的新领域 | <http://new.oversea.cnki.net/KCMS/detail/detail.aspx?dbcode=CJFQ&dbname=CJFD2008&filename=TJSS200806010&uid=WEEvREcwSlJHSldRa1FhdkJkVG1EWmxjVTFCT3ZkL1hKUGkvOVJBNk4ybz0=$9A4hF_YAuvQ5obgVAqNKPCYcEjKensW4IQMovwHtwkF4VYPoHbKxJw!!&v=MDc3NjIxTHV4WVM3RGgxVDNxVHJXTTFGckNVUkxPZmIrUnZGeW5nVkx6UE1TZllmYkc0SHRuTXFZOUVaSVI4ZVg=> |  |
| [Spatial Turn in Narrative Theories-A Summary of Spatial Theories in Narrative] 叙事理论的空间转向——叙事空间理论概述 | <http://new.oversea.cnki.net/KCMS/detail/detail.aspx?dbcode=CJFQ&dbname=CJFD2007&filename=JXSH200711008&uid=WEEvREcwSlJHSldRa1FhdkJkVG1EWmxjVTFCT3ZkL1hKUGkvOVJBNk4ybz0=$9A4hF_YAuvQ5obgVAqNKPCYcEjKensW4IQMovwHtwkF4VYPoHbKxJw!!&v=MDAwNDFyQ1VSTE9mYitSdkZ5bmdXcnpQTHpYWVpyRzRIdGJOcm85RmJJUjhlWDFMdXhZUzdEaDFUM3FUcldNMUY=> |  |
| [Shuli Criticism on the Plots of "The Story of Li Wa"] 《李娃传》情节数理批评 | <http://cn.oversea.cnki.net/kcms/detail/detail.aspx?DbCode=CJFR&dbname=CJFD2013&filename=GLJY201303017&uid=WEEvREcwSlJHSldRa1FhdkJkVG1EWmxjVTFCT3ZkL1hKUGkvOVJBNk4ybz0=$9A4hF_YAuvQ5obgVAqNKPCYcEjKensW4IQMovwHtwkF4VYPoHbKxJw!!> |  |
| [Research on the Evolution of The Tale of Li Wa] 《李娃传》嬗变研究 | <http://new.oversea.cnki.net/KCMS/detail/detail.aspx?dbcode=CJFQ&dbname=CJFD9495&filename=NJDX403.017&uid=WEEvREcwSlJHSldRa1FhdkJkVG1EWmxjVTFCT3ZkL1hKUGkvOVJBNk4ybz0=$9A4hF_YAuvQ5obgVAqNKPCYcEjKensW4IQMovwHtwkF4VYPoHbKxJw!!&v=Mjc0ODZlNEhjL01yb2dxRjU0T2ZnZzV6aEFVNGpoNE9YNlRySDAzZWJPZFJiS2RaK2R2RXlua1VRPT1LeWZQZHI=> |  |
| [Chang'an and Tales in Late Tang Dynasty: Focusing on the Analysis of The Tale of Li Wa] 唐代后期的长安与传奇小说——以《李娃传》的分析为中心 | <https://book.douban.com/subject/1064289/> |  |
| [Automatic Word Segmention of Middle Ancient Chinese Texts with CRFs] 基于CRFs和词典信息的中古汉语自动分词 | <http://new.oversea.cnki.net/KCMS/detail/detail.aspx?dbcode=CJFQ&dbname=CJFDLAST2017&filename=XDTQ201705011&uid=WEEvREcwSlJHSldRa1FhdkJkVG1EWmxjVTFCT3ZkL1hKUGkvOVJBNk4ybz0=$9A4hF_YAuvQ5obgVAqNKPCYcEjKensW4IQMovwHtwkF4VYPoHbKxJw!!&v=MDAwNDFyQ1VSTE9mYitSdkZ5bmhVNy9KUFNuZmY3RzRIOWJNcW85RVpZUjhlWDFMdXhZUzdEaDFUM3FUcldNMUY=> |  |
| [Standard of POS Tag of Contemporary Chinese for CIP (revise)] 信息处理用现代汉语词类标记规范（修订） | <http://new.oversea.cnki.net/KCMS/detail/detail.aspx?dbcode=CPFD&dbname=CPFD9908&filename=JBYW200512001052&uid=WEEvREcwSlJHSldRa1FhdkJkVG1EWmxjVTFCT3ZkL1hKUGkvOVJBNk4ybz0=$9A4hF_YAuvQ5obgVAqNKPCYcEjKensW4IQMovwHtwkF4VYPoHbKxJw!!&v=MDU5NDY3TUkxNFRMeS9TZWJHNEh0VE5yWTlGWmVzS0RoTkt1aGRobmo5OFRuanFxeGRFZU1PVUtyaWZadTl2SGl2a1U3> |  |
| [Dictionary of Tang Chang’an] 唐代长安词典 | <https://book.douban.com/subject/4205523/> |  |
| [A chronicle of Bai Xingjian] 白行简年谱 | <http://new.oversea.cnki.net/KCMS/detail/detail.aspx?dbcode=CJFQ&dbname=CJFD2002&filename=WNXI200203005&uid=WEEvREcwSlJHSldRa1FhdkJkVG1EWmxjVTFCT3ZkL1hKUGkvOVJBNk4ybz0=$9A4hF_YAuvQ5obgVAqNKPCYcEjKensW4IQMovwHtwkF4VYPoHbKxJw!!&v=MDAwNDFyQ1VSTE9mYitSdkZ5bmhVTHZQTWlQVFo3RzRIdFBNckk5RllZUjhlWDFMdXhZUzdEaDFUM3FUcldNMUY=> |  |

## 2.2 Suggestion for cautiously interpreting graphs

***Reviewer’s comment:*** *It is easy to interpret graphs and diagrams, even from formal statistics, yet it is difficult to verify the veracity of these interpretations. It’s not to say, of course, that the interpretation of some graphs is false here, but it would be better to indicate somewhere this call for caution.*

Visualization is powerful, especially for contemporary readers who are used to reading pictures. For this reason in the first version of our manuscript, we used different kinds of visual graphs and diagrams to show one of the possible interpretations by reading the digital representation of texts. However, your valuable suggestion reminds us that the figures are more accurate. So we re-examined the visualized graphs and replaced one with table. Specific values of some parameters are also indicted to in the manuscript to explain the rest figures.

## 2.3 Suggestion for the summary of statistical approaches to literature

***Reviewer’s comment:*** *Note that readers may wish to have a short summary of statistical approaches to literature (without going into details). It would be possible, for example, to quote Franco Moretti's book, Graphs Maps Tree (among others).*

A new review conclusion of the past researches has been added as “Most of these mentioned academic studies are not much easier to read than the classical novel itself. And because of the limitations of perspective and the opacity of personal feelings, it is difficult for readers to synthesize their own experience from previous research.”

A description has been added in the Introduction parts is as follows: “Most of the modern readers are in the digital age when the dialogue between humanities data and computation moves from text digitization, text structuring to text semantics. The representational and interpretive practices to literary texts through quantitative analysis and transmedia argumentation can be traced in Franco Moretti’s ideas about ‘distant reading’ in 2000.”

## 2.4 Suggestion for the explanation of *chronotope*

***Reviewer’s comment:*** *The word "chronotope" comes up 13 times and deserves to be explained at a little more length for readers who do not know Bakhtin.*

Thank you for the reminding. We have given a brief explanation as “This term was adapted by Bakhtin from Einstein's theory of relativity, meaning a spatio-temporal literary unity”

## 2.5 Suggestion for deleting unscientific terms

***Reviewer’s comment:*** *I have no doubt that the text is magnificent (and the authors made me want to read it), but the terms "superb" "splendid" or "shocking" does not sound very scientific. The analysis has nothing to do with the quality of the novel.*

Thank you for the suggestion. We have deleted these terms or substituted with more academic expressions.

## 2.6 Questions about the overall structure of the network in network analysis

***Reviewer’s comment:*** *Do the networks based on a full-text spatially embedded undirected network of characters correspond to small world properties identified by Duncan J. Watts and Steven H. Strogatz (1998) (i.e. small average shortest path length close to purely random graphs and large clustering coefficient significantly higher than expected by random chance) ? if so, what does that tell us about the novel?*

Based on your suggestion, we have listed the parameter files for network analysis in the paper. Our analysis of the overall characteristics of the two network analyses is as following:

1. The characters’ co-occurrence network is a scale-free network, which may prove that the whole story revolves around the male protagonist.

2. The spatially embedded undirected network of characters is a stable small world network, which may prove the setting stages have intentional elements rather than random nor regular.

However, the judgment of network parameters in literature is still a metaphorical category, and it is still expected that more experts will join.

## 2.7 Questions about the English spelling

***Reviewer’s comment:*** *Bakhtin is sometimes misspelled, as well as chronotope. As I am not a native English speaker, I cannot comment on spelling in general.*

Thank you for reminding us and we have revised the spelling mistakes.

# 3. Responses to suggestion of reorganizing the paper from Reviewer #2

Thank the reviewers#2 for your suggestions on this paper, which inspires us to rethink the whole structure of the article.

## 3.1 About research question and paper’s narrative

***Reviewer’s comment:*** *In this sense, I suggest the author reorganize the narrative and structure of the paper, highlighting the research question and novelty of the paper. Rather than solely demonstrating the toolbox, I hope the author could provide the insight of each analysis.*

*I have several questions regarding the “research question” of the paper and some technical details of the methods.*

*First, despite several methods have been adopted and hundreds of analysis have been made, it is very difficult to find a very clear statement of the research question in the paper. Is it about a demonstration of a new method? (It seems not, since all methods have been invented before). Or is it about using these analysis to support a known theory? Or the authors want to present some new insights from the analysis? The authors may have a clear answer to these questions and may have some deep insights in their mind, but it is very difficult (at least for me) to find the answer in the current presentation of the work. The current version is so over overwhelmed with different analysis without a clear thread to link them. Without a key message, it is also hard for the readers to tell the purpose of each analysis and understand the relationship between them. …*

*In this sense, I suggest the author reorganize the narrative and structure of the paper, highlighting the research question and novelty of the paper. Rather than solely demonstrating the toolbox, I hope the author could provide the insight of each analysis.*

We had hoped to organize a framework of sets of digital models that integrate literary theory, thus providing contemporary readers with an additional opportunity to understand Chinese classical novels more appropriately. But the spatio-temporal representation in the last manuscript did not pay much attention to the construction of the overall logic. Actually we were a bit cautious about reorganizing the narrative from the author's perspective in the last version since we wanted to provide readers with the freedom to read in-depth, which however made it difficult to read our paper.

Your suggestion is very valuable. In this revised manuscript, we decided to show one of the possibilities under this framework of sets of digital models that integrate literary theory. It is a knowledge model or narrative to illustrate the theme "what-how-why" about the growth of the male protagonist based on the our team’s experience. This knowledge model is gradually obtained through in-depth analysis (we have made more in-depth interpretation of diagrams and there do exist some new insights which we choose not to highlight) and concatenation of various diagrams in a logical loop of "Time-Space-time-Space".

## 3.2 About different community detection algorithms

***Reviewer’s comment:*** *For example, what is the message the author want to convey in Fig. 17? What is the new insight of the plot? How could that deepen the reader’s understanding of the book since it is like a confirmation that the main character is Zheng? By the way, is the color representing the communities learned from the network, did you use the same method shown in Fig. 21? If a different modularity method is adopted (say Modularity Q proposed by Girvan and Newman), whether the results shown in these two figures still hold?*

Different algorithms may indeed lead to different answers, especially for community detection algorithm of network analysis, because communities have relatively vague boundaries. However, this modularity analysis at the "space" level is relatively in line with the more close analysis in the "time" and "space-time" level. Therefore we think it has some kind of credibility. But we do not deny the interpretation nature of this digital representation, which is a problem we will not or not able to avoid. We just show a possibility.

## 3.3 About the assignment of the sentiment orientation (so) value

***Reviewer’s comment:*** *The assignment of the sentiment orientation (so) value seems a little bit arbitrary, since several words and characters could have ambiguous meaning and it is possible for different people have different feelings about the same word. Could the authors provide some cross-validations of the value assignment to make sure the value indeed present the basic expression? For example, maybe the authors could borrow tools from network science (as they did in the paper), and build a network of the sentiment expressions? (if two sentiment expressions are shown in one sentence or from one reading time snapshot, a link will be added between them) Of course, the author could try other alternative methods to do the cross-validation.*

We actually have done two rounds of SO assignment. The percentage of consent of two rounds of SO value assignment is >0.9.

## 3.4 Questions about non-standard cartography

***Reviewer’s comment:*** *Why the compass also have a color of light blue? Does that mean the person has shown there? The same issue for the lower panel of Fig. 13.*

We have narrowed the boundary of IDW interpolation analysis.

## 3.5 Questions about details of measuring the “stratum”

***Reviewer’s comment:*** *Can the authors provide the details of how do they measure the “stratum” in Fig. 18?*

We replace the interpolation analysis of social stratum with a statistical chart, and count the social stratum of characters in each place, weighted by the number of times characters enter places. The classification of social stratum in the story from untouchable to nobles is as follows: (1) level 1: beggar, servant, and sex worker; (2) level 2: businessman, civilian, madam; (3) level 3: ward head (里胥), candidate student, successful candidate, county judicial official (贼曹); and (4) level 4: Chang’an officials (京尹) and officials from other places.

## References:

Bakhtin MM. Forms of Time and of the Chronotope in the Novel: Notes toward a Historical Poetics. In: Richardson B, editors. Narrative dynamics: Essays on time, plot, closure, and frames. Columbus: The Ohio University; 2002. pp. 15-24.

Zoran G. Towards a Theory of Space in Narrative. Poetics Today. 1984; 5(2): 309-335.

Mitchell WJT. Spatial Form in Literature: Toward a General Theory. Critical Inquiry. 1980; 6(3): 539-567.
