# Supplementary material for: Representation of the spatio-temporal narrative of The Tale of Li Wa李娃传
Source: PLoS One. 2020 Apr 23;15(4):e0231529. doi: 10.1371/journal.pone.0231529 (PMC7180071; doi:10.1371/journal.pone.0231529)
Supplement: Attachment — Submitted filename: Response to Reviewers.doc [file pone.0231529.s014.doc]

# Responses to Reviewers

# 1. Response to the “writing and presentation style” issue noted by the editor

***Editor’s comment:*** *I agree with Reviewer 1 regarding writing and presentation style of the manuscript. There are a few places that can be better presented.*

We have revised the writing and presentation style of the manuscript to the best of our capability and the manuscript has revised by a professional language editor. Further, we have explained and revised some technical details of the methods, including the addition of another community detection algorithm, supplementary statistics regarding emotion-related linguistic rules, and an explanation of the network’s degree distribution.

# 2. Responses to the suggestion of algorithms and analysis from Reviewer #1

Thank you for your suggestions regarding the technical details of the methods, which have inspired us to reexamine the methods and make some necessary revisions.

## 2.1 About community detection method

***Reviewer’s comment:*** *About the community detection method (Answer 3.2). I am still expecting some explanations of the insights of the figure. Did it change our previous understanding of the narrative structure of the work? Or it only confirms that Zheng is the main character?*

*And I still recommend the authors to try other algorithms of community detection (e.g. Modularity Q, etc.). I fully understand that here the author only presents one possibility, and results obtained from other algorithms could be very different. But the authors should report the differences in their paper and let the reader know there ARE other possibilities and only one possibility is presented here.*

Because the characters’ co-occurrence network is not a large-scale network, based on your recommendation, it seemed advisable to attempt other divisive algorithms such as Girvan–Newman (Girvan and Newman, 2002) for a hierarchical community structure rather than the agglomerative algorithm alone (they are both based on modularity). In general, there is no difference between the two algorithms, although the former is slightly more accurate because it involves a top-down approach. Nonetheless, our data show an interesting phenomenon: the conflict of characters in the top-down model (Fig 1 below) seems to be much weaker than that in the bottom-up model (Fig 2), although the overall structure of the two is similar. According to Fig 1, Zheng’s father and Zheng are from the same class, whereas Fig 2 shows Zheng’s embarrassing situation between his father and Li Wa, which is somewhat similar to the different views proposed by scholars with different perspectives. However, it should be noted that the co-occurrence network of characters is not a real social network in the novel. In actuality, many characters around Zheng such as the predecessor have potential social relations with others. We have included further discussion concerning the topic in our manuscript in lines 538-547.

a

b

**Fig 1. Statistics of characters in co-occurrence network, cluster and betweenness centrality in a. new Girvan–Newman** **divisive algorithm for community detection**

**Fig 2. Statistics of characters in co-occurrence network, cluster and betweenness centrality in original agglomerative algorithm for community detection, use edge weights off**

We also tested the spatially embedded network of characters. The results of top-down model (Fig 3) seem to be a slightly more complex to explain from the perspective of literary geography than the bottom-up model (Fig 4), although there are many similarities between the overall structures of the two. Therefore, we decided not to include the new model’s results in the manuscript, as this may require a considerable amount of sources or data for a comparative study of literary-geographical phenomena.

**Fig 3. Network analysis of characters and places in Girvan–Newman clustering analysis and weighted degree centrality based on a full-text,** **spatially embedded, undirected network of characters.**

**Fig 4. Network analysis of characters and places in modularity class analysis (use edge weights on) and weighted degree centrality based on a full-text, spatially embedded, undirected network of characters.**

## 2.2 About the assignment of the sentiment orientation (SO) value

***Reviewer’s comment:*** *About the sentiment orientation (SO) value (Answer 3.3). The authors state that they have done two rounds of SO assignment, which I fully appreciate. But this is more like a robustness test of the convergence of the method, instead of a validation of the method itself. I am still wondering that, like what I said in the previous comments, did the SO value indeed present the basic expression? Could the authors present more detailed information or empirical evidence of this point?*

Thank you for your suggestion on sentiment analysis. We have included further discussion concerning the topic in our manuscript in lines 179-184. We take the algorithm of “document sentiment classification using sentiment lexicons*”* (Liu, 2015) to calculate the emotional curve (the trajectory of the integral function of SO value) in the framework of time and space. In terms of the sentiment lexicon generation, there are three main approaches: a manual approach, a dictionary-based approach, and a corpus-based approach. However, the latter two methods are based on the first, which exploit some linguistic rules or conventions based on manually-collected seed sentiment words to identify other sentiment words (Liu, 2015). Because there are no extant and ready-made sentiment lexicons, we use manual assignment for the sentiment lexicon. The artificial test of emotion curve regarding the plot’s ups and downs in our manuscript also proves that our assignment and the algorithm that is related to sentiment have some advantages.

As mentioned in the manuscript (in Word-level analysis), the linguistic rules of ancient Chinese are considerable different from those of modern Chinese, and we cannot determine the rules that should be used here to measure the accuracy of the manual assignment of SO. Nevertheless, based on your suggestion, we have made an interesting attempt, assuming that the sentiment words in ancient Chinese follow the same logic for current sentiment analyses in that sentiment words have context-dependent orientations, i.e., the total distance among words with the same orientation sentiment expression is closer than that among different ones (Turney et al., 2003, Kamps et al., 2004).

Based on the unigrams of *The Tale of Li Wa* removing stop words, we complete the training of its word2vec model with gensim package (parameters: sg = 0, size = 50, window = 5, min_count = 1, iter = 20) and acquire the correlation (*cosine_similarity*, −0.4 ~ 1) between each two words––the closer the value is to 1, the higher the correlation (based on vector distance) between them (Rehurek et al., 2010). Consequently, we take this correlation between word A (*Source*) and word B (*Target*) multiplied by the SO value of word B as the weight of the edge, establishing a sentiment network of words in Gephi. Gephi’s calculated *weighted outdegree* of each word reflects its relationship with all other sentiment words, i.e., the larger the *weighted outdegree* a word is, the more positive its sentiment is, and vice versa. However, the results of different type of manually-collected sentient words (1, **−**1, 0) reveal that the ancient Chinese may not simply conform to the hypothesis that the sentiment words have context-dependent orientations (Fig 5.a–c), as our SO assignment is not so outrageous. This part has been simply added to the manuscript in the part of “Word-level analysis”, as well as to my Github project (<https://github.com/aayi/The-Tale-of-Li-Wa>).

**a**

**b**

**c**

**Fig 5. Weighted outdegree distribution of words’ sentiment network (a. SO = 1, b. SO = −1, c. SO = 0).**

## 2.3 About the scale-free network

***Reviewer’s comment:*** *In line 532, the authors added that the network is “a small-sized scale-free network”, they said “a few nodes known as the hubs that have extremely many connections/edges”. But it seems to me that the largest node in the network only has a degree around 10 to 20, which makes it very hard to be a scale-free network. Did I miss something? Did the authors do some statistics test about this statement?*

**Figure 6. Degree distribution.** **Figure 7. Degree distribution (ln-ln).**

We have reconfirmed the characters’ co-occurrence network whose degree distribution turns out to asymptotically follow a power law (Figs 6–7). It is very likely a power-law network with scale-free property, although it is not a large-scale network (汪小帆et al., 2012, 郭世泽et al., 2012). However, to be rigorous, we decided to revise the phrasing in the manuscript (lines 532–533) to “a probable small-scale power-law network.”

# 3. Responses to Reviewer #2

Thank you for your suggestions regarding our paper.

## 3.1 About the new reference

***Reviewer’s comment:*** *A new reference that might be of interest (?): In his analysis of the book L'Éducation sentimentale (in La Distinction. Critique sociale du jugement, 1979), Bourdieu used a map of the Latin Quarter (i.e. Quartier Latin, in Paris) and gives a relational reading: he shows the places of geographical (and therefore social) origin of the different protagonists and makes it possible to point out positions that take on a large surplus of meaning when we oppose / put them in relationship with each other.*

This new reference has reminded us to include further supplements to the field of the literary cartography as a quantitative and transmedia approach to literary geography in our manuscript–– “…can be traced in Franco Moretti’s … and his practice of literary cartography integrating “spatial criticism” in 1999” (lines 125–126).

## 3.2 Questions on spelling

***Reviewer’s comment:*** *About question 5, not being a native English speaker, I cannot judge the spelling of the paper.*

Thank you for the reminder; we have made revisions within the manuscript with respect to language and grammar to improve accuracy and readability.

## References

Girvan M, Newman MEJ. Community structure in social and biological networks. Proceedings of the National Academy of Sciences. 2002 Jun; 99(12): 7821-7826. doi: 10.1073/pnas.122653799.

Kamps J, Marx M, Mokken RJ, et al. Using WordNet to measure semantic orientation of adjectives. In: Maria TL, Maria FX, Fátima F, Rute C, Raquel S, editors. Proceedings of the Fourth International Conference on Language Resources and Evaluation; 2004 May; Lisbon: European Language Resources Association, 2004. pp.1115-1118.

Liu B. Sentiment analysis: Mining opinions, sentiments, and emotions. New York: Cambridge University; 2015.

Rehurek R, Sojka P. Software framework for topic modelling with large corpora. In: Rene W, Hamish C, Jon P, Elena B, Ekaterina B, Udo H, Karin V, Anni RC, editors. Proceedings of the LREC 2010 Workshop on New Challenges for NLP Frameworks; 2010 May 22; Valletta: ELRA; 2010. pp. 45-50.

Turney PD, Micharel LL. Measuring praise and criticism: Inference of semantic orientation from association. ACM Trans Inf Syst. 2003; 21(4): 315-46.

汪小帆, 李翔, 陈关荣. [Network science: An introduction] 网络科学导论. Beijing: Higher Education Press; 2012. Chinese.

郭世泽，陆哲明. [The basic theory of complex network] 复杂网络基础理论. Beijing: Science Press; 2012. Chinese.
