# Supplementary material for: Representation of the spatio-temporal narrative of The Tale of Li Wa李娃传
Source: PLoS One. 2020 Apr 23;15(4):e0231529. doi: 10.1371/journal.pone.0231529 (PMC7180071; doi:10.1371/journal.pone.0231529)
Supplement: Attachment — Submitted filename: Response to Reviewers.doc [file pone.0231529.s015.doc]

# Responses to Editors and Reviewers

# 1. Response to the “small scale free network” issue noted by the editor and the reviewer

***Editor’s comment:*** *I agree with the reviewer's comment about the claim of having analysed a "**small scale free network". If the authors believe it is a very relevant point to discuss and to support, they can attempt more advanced interpolation techniques as suggested by the reviewer, an submit an improved version of the fit, which however in my opinion would probably make very little sense anyhow, see https://science.sciencemag.org/content/335/6069/665*

*My suggestion here is just to avoid specifying "scale free network" in the paper, if possible, limiting the statement to something on the line of "a few nodes known as the hubs that have extremely many connections/edges". If this correction is promptly done, I would be able to accept the paper without further review.*

***Reviewer’s comment:*** *Based on the degree distribution plot they showed, I am still not quite convinced that it is a scale-free network, or "Power-law network" as they phrased. I suggest the author to do one more statistical test following the paper "power law distributions in empirical data" by Aaron Clauset et al., where they have provided a rather accurate way to test a distribution is power law or not. Their codes are public available online.*

Thanks for the reviewer’s comment and the editor’s advice. We have followed the editor's advice and revised the manuscript as follows: “Character statistics enable a network analysis of the characters’ co-occurrence network in the entire document in Fig 17 and 18 (see S7 File), which indicates a network around hubs (a few nodes have much more connections/edges).” (lines 531–533)

# 2. Responses to the laboratory protocols from the editor

Thank you for your suggestions, we have included the DOI link (http://dx.doi.org/10.17504/protocols.io.bdyni7ve) in the Materials and methods section of our manuscript.

# 3. Other problems

We have also revised other minor errors in the manuscript.
